# Stability of genomic imprinting in human induced pluripotent stem cells

**DOI:** 10.1186/1471-2156-14-32

**Published:** 2013-04-30

**Authors:** Hitoshi Hiura, Masashi Toyoda, Hiroaki Okae, Masahiro Sakurai, Naoko Miyauchi, Akiko Sato, Nobutaka Kiyokawa, Hajime Okita, Yoshitaka Miyagawa, Hidenori Akutsu, Koichiro Nishino, Akihiro Umezawa, Takahiro Arima

**Affiliations:** 1 Department of Informative Genetics, Environment and Genome Research Center, Tohoku University Graduate School of Medicine, 2-1 Seiryo-cho, Aoba-ku, Sendai, 980-8575, Japan; 2 Department of Reproductive Biology, National Research Institute for Child Health and Development, 2-10-1 Ohkura Setagaya-ku, Tokyo, 157-8535, Japan; 3 Research team for Geriatric Medicine (Vascular Medicine), Tokyo Metropolitan Institute of Gerontology, 35-2 Sakaecho, Itabashi-ku, Tokyo, 173-0015, Japan; 4 Laboratory of Veterinary Biochemistry and Molecular Biology, Faculty of Agriculture, University of Miyazaki, 1-1 Gakuen-Kibanadai-Nishi, Miyazaki, 889-2192, Japan

**Keywords:** Genomic imprinting, Loss of imprinting (LOI), DNA methylation, Histone modification, Human induced pluripotent cells

## Abstract

**Background:**

hiPSCs are generated through epigenetic reprogramming of somatic tissue. Genomic imprinting is an epigenetic phenomenon through which monoallelic gene expression is regulated in a parent-of-origin-specific manner. Reprogramming relies on the successful erasure of marks of differentiation while maintaining those required for genomic imprinting. Loss of imprinting (LOI), which occurs in many types of malignant tumors, would hinder the clinical application of hiPSCs.

**Results:**

We examined the imprinting status, expression levels and DNA methylation status of eight imprinted genes in five independently generated hiPSCs. We found a low frequency of LOI in some lines. Where LOI was identified in an early passage cell line, we found that this was maintained through subsequent passages of the cells. Just as normal imprints are maintained in long-term culture, this work suggests that abnormal imprints are also stable in culture.

**Conclusions:**

Analysis of genomic imprints in hiPSCs is a necessary safety step in regenerative medicine, with relevance both to the differentiation potential of these stem cells and also their potential tumorigenic properties.

## Background

Human induced pluripotent stem cells (hiPSCs) represent a promising therapeutic tool for many diseases, and might be useful for regenerating aged tissues and organs at high risk of failure [1,2]. However, the intrinsic self-renewal and pluripotency of hiPSCs potentially make them tumorigenic, hindering their clinical application [3-5]. hiPSCs are generated through epigenetic reprogramming of somatic tissue. It was initially thought that hiPSCs and human embryonic stem cells (hESCs) shared a high degree of epigenetic similarity [6,7]. However, recent reports have indicated that substantial differences exist between hiPSCs and hESCs with regard to gene expression, miRNA expression and DNA methylation [8-10]. Cell-of–origin-specific genetic and epigenetic differences exist in hiPSCs [11] and some of these stem cell lines spontaneously differentiate during serial passage [12]. Extensive evaluation of hiPSCs is consequently an essential component of the process required for their safe use in regenerative medicine.

Many types of malignant tumors are characterized by complex genetic and epigenetic alterations, including loss of heterozygosity (LOH) and loss of imprinting (LOI) [13,14]. Such alterations are presumed to represent the second hit, according to Knudson’s two-hit hypothesis (OMIM #167000) [15]. However, alterations in DNA methylation can also occur as the first hit during human carcinogenesis [16]. Alterations in the expression of imprinted genes represent one of the most common changes seen in cancer [17,18]. Some imprinted genes, including *H19*[19], *GTL2*[20], *PEG1, PEG3*[21], *LIT1* (*KCNQ1OT1*) [22] and *ZAC*[23] are known to act, or are strongly implicated to act, as tumor suppressor genes (TSGs). Furthermore, imprinted genes play key roles in regulating growth and differentiation [24]. Thus the aberrant expression of imprinted genes may contribute to tumorigenesis or alter the differentiation potential of stem cells.

The monoallelic expression of imprinted genes is reliant on epigenetic mechanisms, most notably DNA methylation, which is established in the male and female germlines at discrete locations termed germline or gametic differentially methylated regions (gDMRs) [25]. Imprinted domains generally contain several genes displaying allele-specific expression and gDMRs within these domains act as imprinting centers or imprint control regions for the domain [26]. The majority of imprinted genes reside within these complex domains [27]. Although gametic DMRs are maintained throughout the life of the organism, genes within the domain can be imprinted in tissue- and developmentally specific manners [28].

In a recent paper, we demonstrated that hiPSCs exhibit epigenetic patterns distinct from hESCs [29]. After continuous passaging of the hiPSCs, these differences diminished such that over time the hiPSCs more closely resembled hESCs. However, we found that the imprinted DMRs showing abnormal methylation in early passage hiPSCs did not resolve during passaging. In this study we focused on the expression of imprinted genes in hiPSCs. Several reports on imprinted gene expression in hESCs demonstrate a substantial degree of instability [30]. Less is known regarding the stability of imprints in hiPSCs, although some work has begun [31]. We are particularly concerned with the stability of imprints in pluripotent stem cells during prolonged culture. Here, we examined the imprinting status and expression levels of eight imprinted genes and the methylation status of their DMRs in five independently derived hiPSCs. We found that the frequency LOI was very low in the early passaged lines. We also found that, in contrast, the epigenetic changes that took place at non-imprinted loci during prolonged culture for both normal and aberrant imprints were stably inherited despite prolonged passaging of the lines.

## Results

### Loss of heterozygosity (LOH) and loss of imprinting (LOI) in hiPSCs

We first determined whether hiPSCs showed LOH by comparing the restriction fragment length polymorphism (RFLP) patterns of the original tissue DNA with those of the hiPSC DNA samples. Samples in which RFLPs were present in the original DNA sample but absent or with an altered ratio in the hiPSC samples were considered to exhibit LOH. We found no evidence for LOH at the 8 loci tested (*H19*, *IGF2*, *KCNQ1*, *LIT1*, *GTL2*, *PEG1*, *PEG3* and *NDN*).

**Figure 1:**
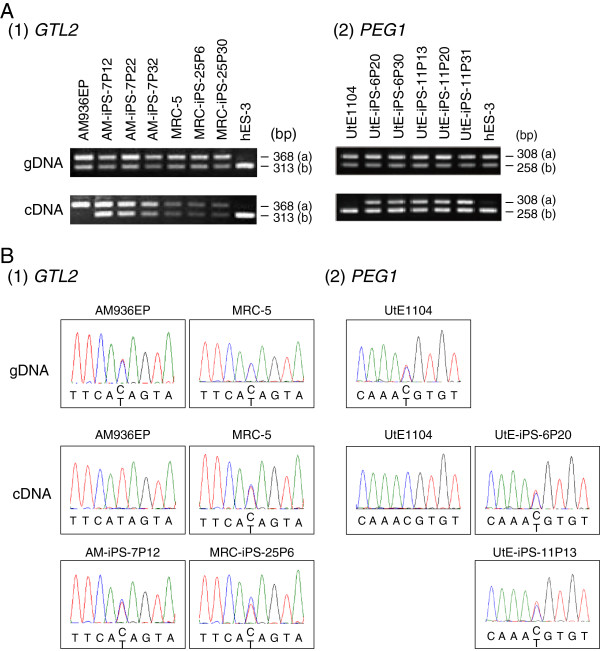
**Loss of imprinting in hiPSCs.** Loss of allelic expression of *GTL2* in cell line AM-iPS-7 (1) and *PEG1* in cell lines UtE-iPS-6 and 11 (2). PCR products amplified from paired genomic DNA and cDNA samples were digested with the specified restriction enzymes (**A**). Results were confirmed by direct sequencing (**B**). AM936EP and UtE1104 are primary culture cells made from amniotic membrane and uterine endometrium cells, respectively. Band ‘a’: without a restriction site. Band ‘b’: with a restriction site.

We next performed RT-PCR and RFLP analyses to identify samples that demonstrated loss of imprinting (LOI). Where expression of genes was low in undifferentiated cells, it was not possible to determine their imprinting status (*H19* in MRC-iPS and UtE-iPS, *IGF2* in PAE-iPS and *GTL2* in Edom-iPS). Of the 16 informative loci, we identified LOI at three loci in hiPSCs, *GTL2, PEG1* and *PEG3,* but we did not detect any LOI in hESCs (Table 1). Of particular interest, we observed loss of imprinting during the process of establishing the AM-iPSC (*GTL2)* and UtE-iPSC (*PEG1*) lines (Figure 1A). Where LOI was observed in early passage cells, this was maintained even after 30 or more passages (Figure 1).

**Table 1:** LOI and MOI in hiPSCs

		**H19**		**IGF2**		**PEG3**		**PEG1**		**GTL2**		**KCNQ1**		**NDN**		**LIT1**	
		(RsaI)		(ApaI)		(MnlI)		(AflIII)		(TaaI)		(SmaI)		(MboI)		(RsaI)	
Cell	Passage	gDNA	cDNA	gDNA	cDNA	gDNA	cDNA	gDNA	cDNA	gDNA	cDNA	gDNA	cDNA	gDNA	cDNA	gDNA	cDNA
AM936EP	P9	a/b	a	b	-	a	-	b	-	a/b	a	a	-	b	-	a/b	a
AM-iPS -2	P13	a/b	a	b	-	a	-	b	-	a/b	a	a	-	b	-	a/b	a
AM-iPS -2	P19	a/b	a	b	-	a	-	b	-	a/b	a	a	-	b	-	a/b	a
AM-iPS -2	P35	a/b	a	b	-	a	-	b	-	a/b	a	a	-	b	-	a/b	a
AM-iPS -3	P9	a/b	a	b	-	a	-	b	-	a/b	a	a	-	b	-	a/b	a
AM-iPS -3	P21	a/b	a	b	-	a	-	b	-	a/b	a	a	-	b	-	a/b	a
AM-iPS -3	P29	a/b	b	b	-	a	-	b	-	a/b	a	a	-	b	-	a/b	a
AM-iPS -3	P36	a/b	a	b	-	a	-	b	-	a/b	a	a	-	b	-	a/b	a
AM-iPS -7	P12	a/b	a	b	-	a	-	b	-	a/b	a/b	a	-	b	-	a/b	a
AM-iPS -7	P22	a/b	a	b	-	a	-	b	-	a/b	a/b	a	-	b	-	a/b	a
AM-iPS -7	P32	a/b	a	b	-	a	-	b	-	a/b	a/b	a	-	b	-	a/b	a
AM-iPS -8	P13	a/b	a	b	-	a	-	b	-	a/b	a	a	-	b	-	a/b	a
AM-iPS -8	P20	a/b	a	b	-	a	-	b	-	a/b	a	a	-	b	-	a/b	a
AM-iPS -8	P37	a/b	a	b	-	a	-	b	-	a/b	a	a	-	b	-	a/b	a
AM-iPS -20	P8	N.T.	a	N.T.	-	N.T.	-	N.T.	-	N.T.	a	N.T.	-	N.T.	-	N.T.	a
AM-iPS -20	P11	a/b	a	b	-	a	-	b	-	a/b	a	a	-	b	-	a/b	a
AM-iPS -20	P14	a/b	a	b	-	a	-	b	-	a/b	a	a	-	b	-	a/b	a
AM-iPS -20	P16	a/b	N.D.	b	-	a	-	b	-	a/b	a	a	-	b	-	a/b	a
AM-iPS -20	P32	a/b	N.D.	b	-	a	-	b	-	a/b	a	a	-	b	-	a/b	a
PL551Ar	P16	a/b	a	a/b	a	b	-	a/b	N.D.	b	-	b	N.D.	a	-	a	-
PAE-iPS -05	P19	a/b	a	a/b	N.D.	b	-	a/b	N.D.	b	-	b	-	a	-	a	-
PAE-iPS -05	P31	a/b	a	a/b	N.D.	b	-	a/b	N.D.	b	-	b	-	a	-	a	-
PAE-iPS -11	P14	a/b	a	a/b	N.D.	b	-	a/b	N.D.	b	-	b	-	a	-	a	-
PAE-iPS -11	P18	a/b	a	a/b	N.D.	b	-	a/b	N.D.	b	-	b	-	a	-	a	-
PAE-iPS -11	P30	a/b	a	a/b	N.D.	b	-	a/b	N.D.	b	-	b	-	a	-	a	-
MRC-5	-	a/b	N.D.	b	-	a	-	b	-	a/b	a/b	a/b	N.D.	a/b	N.D.	a	-
MRC-iPS -16	P30	a/b	N.D.	b	-	a	-	b	-	a/b	ND	a/b	b	a/b	a	a	-
MRC-iPS -25	P6	a/b	N.D.	b	-	a	-	b	-	a/b	ND	a/b	b	a/b	a	a	-
MRC-iPS -25	P30	a/b	N.D.	b	-	a	-	b	-	a/b	ND	a/b	b	a/b	a	a	-
MRC-iPS -40	P11	a/b	N.D.	b	-	a	-	b	-	a/b	ND	a/b	b	a/b	a	a	-
MRC-iPS -40	P30	a/b	N.D.	b	-	a	-	b	-	a/b	ND	a/b	b	a/b	a	a	-
UtE1104	P9	a/b	N.D.	a	-	a/b	a/b	a/b	b	a/b	a/b	b	N.D.	b	-	a	-
UtE-iPS -6	P20	a/b	N.D.	a	-	a/b	a/b	a/b	a/b	a/b	a/b	b	-	b	-	a	-
UtE-iPS -6	P31	a/b	b	a	-	a/b	a/b	a/b	a/b	a/b	a/b	b	-	b	-	a	-
UtE-iPS -11	P13	a/b	N.D.	a	-	a/b	N.D.	a/b	a/b	a/b	a	b	N.D.	b	-	a	-
UtE-iPS -11	P20	a/b	N.D.	a	-	a/b	N.D.	a/b	a/b	a/b	a/b	b	N.D.	b	-	a	-
UtE-iPS -11	P30	a/b	N.D.	a	-	a/b	N.D.	a/b	a/b	a/b	a	b	-	b	-	a	-
Edom22	P5	b	-	a/b	a/b	a	-	a/b	b	b	-	a/b	a	a	-	a	-
Edom-iPS -1	P27	b	-	a/b	N.D.	a	-	a/b	b	b	-	a/b	a	a	-	a	-
hES 3	P29	a/b	a	b	-	a/b	a	a/b	b	b	-	a	-	a/b	b	a	-
SEES 1	P10	a/b	a	a/b	a	a	-	b	-	a/b	b	a	-	a	-	a	-
SEES 4	P9	a/b	b	a/b	a	a	-	b	-	b	-	a	-	a/b	b	a	-

A summary of LOI and MOI RFLP data for the 8 imprinted genes analyzed in 22 hiPSCs and 3 control hES cell lines. hiPSCs derived from extraembryonic amniotic membrane (AM-iPS), embryonic lung tissue (MRC-iPS), uterine endometrium (UtE-iPS), adult menstrual blood (Edom-iPS) and extraembryonic placental tissue (PAE-iPS). Samples were analyzed at the specified passage number. (-): not informative.

### Expression level of the imprinted genes in hiPSCs

**Figure 2:**
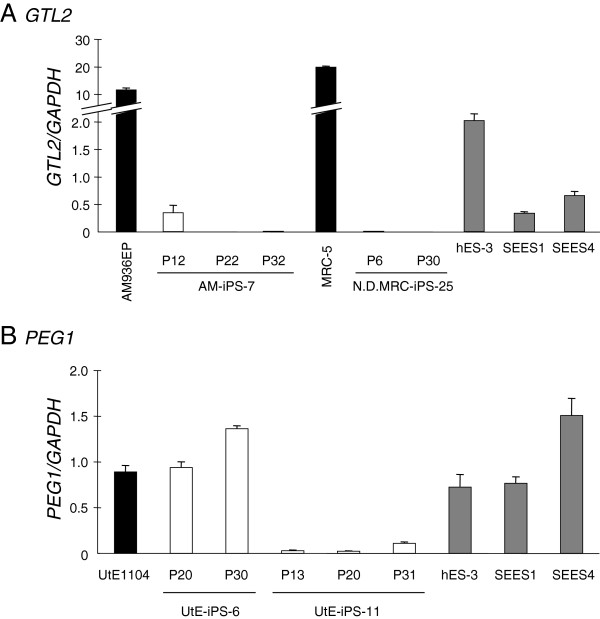
**Reduced expression of hiPSCs with LOI.** Gene expression levels of *GTL2* (**A**) and *PEG1* (**B**) in the original and the passaged hiPSCs were compared to that of hESCs. The *GAPDH* ratio was calculated. The bars indicate the means ± standard deviation (SD) from two replicates. N.D.: not determined.

LOI can refer to silencing of an originally active allele or expression of a normally silent allele. Therefore, we compared the expression levels of the three genes that displayed LOI in hiPSCs and hESCs (Table 1). The expression of *IGF2* and *GTL2* was decreased in almost all the hiPSC lines in comparison with the hES cells (Additional files 1 and 2). *GTL2* in cell line AM-iPS-7 and *PEG1* in line UtE-iPS-11 showed apparent biallelic expression but their expression levels were relatively low in comparison to hESCs with reduced expression maintained stably through to late passages (Figure 2). In contrast, expression of *PEG1* in cell line UtE-iPS-6 was not significantly different from that of hESCs. These results were in accordance with the DNA microarray analysis data we already reported [29]. Since, in two cases, LOI correlated with reduced gene expression, this has potential functional implications due to loss of function.

### Analysis of the DNA methylation status and the histone modification of GTL2 and PEG1 DMRs in hiPSC lines

**Figure 3:**
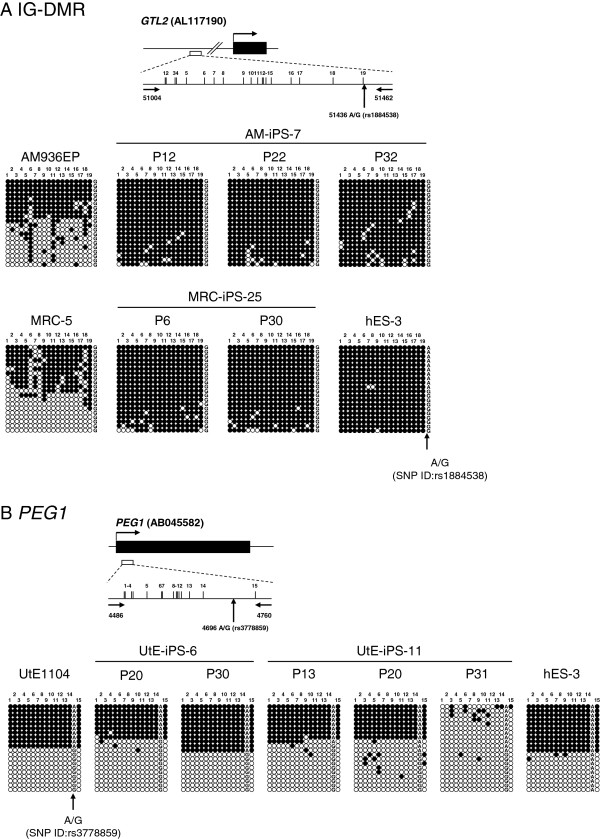
**Aberrant DNA methylation of hiPSCs with LOI.** Bisulfite PCR sequencing methylation assay of genomic DNA prepared from AM-iPS-7 and MRC-iPS-25 at the IG-DMR (*GTL2*-DMR) (**A**) and UtE-iPS-6 and 11 at *PEG1* (**B**). Each row represents a unique methylation profile within the pool of 20 clones sequenced. Closed and open circles represent methylated and unmethylated CpGs, respectively. The numbers represent the percentages of methylation by bisulfite sequencing. SNPs are shown by arrows.

**Figure 4:**
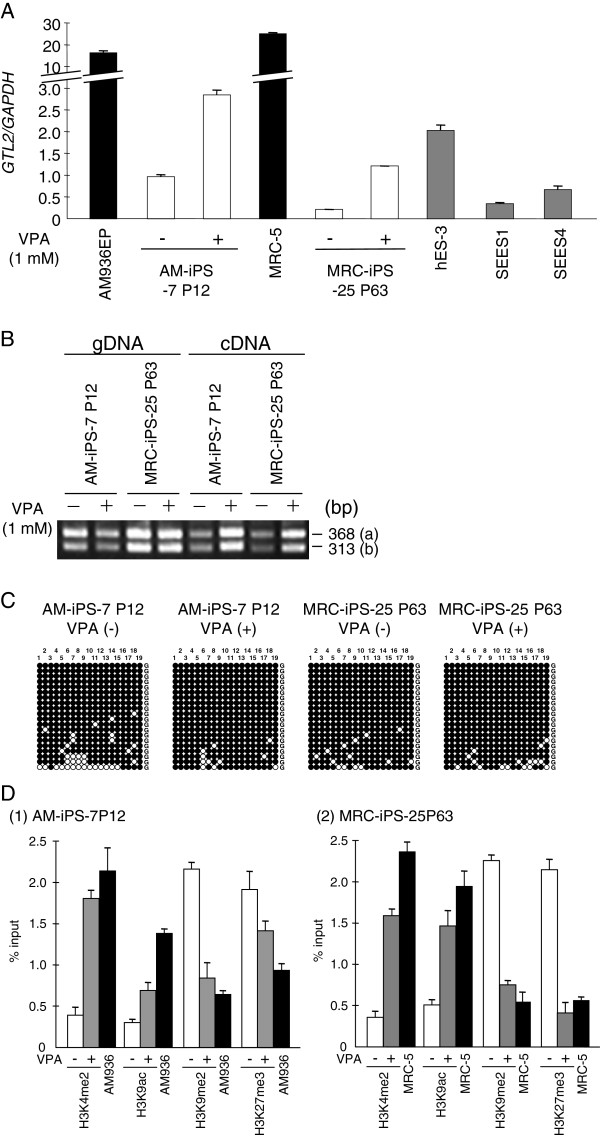
**Reactivation of *GTL2 *expression by treatment with VPA.** Reactivation of *GTL2* expression by treatment with VPA (**A**). The expression level of *GTL2* mRNA was restored by VPA treatment. Gene expression of the original cells and of the hiPSCs was compared to that of hESCs. The *GAPDH* ratio was calculated. The bars indicate the means ± SD from two replicates. The imprinting status of *GTL2* was stable in response to VPA (**B**). Methylation status in bisulfite-PCR sequencing analyses of IG-DMR is unchanged (**C**). Histone modifications of the *GTL2* promoter were changed by VPA (D). The immunoprecipitation/input ratio was calculated. The bars indicate the means ± SD from three replicates.

We determined the allele-specific methylation status of the *GTL2* (IG-DMR) and *PEG1* imprinted DMRs using polymorphic bisulfite-PCR sequencing (Figure 3). In cell line AM-iPS-7, which showed LOI and reduced expression of *GTL2,* we observed hypermethylation of IG-DMR, which was maintained during continuous passaging. IG-DMR methylation is normally present on the silent allele of *GTL2*[32], which suggests aberrant signaling between this DMR and *GTL2* expression. In cell line UtE-iPS-11, in which there was LOI and reduced expression of *PEG1,* abnormal methylation was detected in passage 31 cells but not earlier passages. In cell line UtE-iPS-6, in which there was LOI but not reduced expression of *PEG1,* abnormal methylation was not detected. Allele-specific expression of some genes has been reported to be regulated by histone modification rather than direct DNA methylation [33-35]. We therefore analyzed histone modifications in the hiPS cell line by chromatin immunoprecipitation (ChIP) analyses using the following antibodies: dimethylated H3-Lys4 (H3K4me2), acetylated H3-Lys9 (H3K9ac), H3K9me2, and H3K27me3. H3K4me2 and H3K9ac mark active genes and H3K9me2 and H3K27me3 are repressive marks. In the *GTL2* promoter region, H3K9me2 and H3K27me3 were enriched in AM-iPS-7 and MRC-iPS-25 cells (Figure 4D).

#### Reactivation of the imprinted genes by the HDACi treatment

Previous reports demonstrated that the *GTL2* gene was aberrantly silenced in most mouse iPSC lines but that expression could be restored through HDACi treatment [36,37]. In our study, AM-iPS and MRC-iPS cells showed LOI of *GTL2*, with a reduction in gene expression and hypermethylation of the IG-DMR. To assess whether *GTL2* expression could be restored, AM-iPS and MRC-iPS cells were treated with the HDAC inhibitor VPA (sodium valproate). VPA-treated cells did achieve a 3.0–5.8-fold increase in *GTL2* expression levels (Figure 4A) and H3K4me2 and H3K9ac were enriched in its promoter region (Figure 4D). However, the DNA methylation pattern was stable under VPA treatment and the imprinting status of *GTL2* was not changed, with cells maintaining biallelic expression of the gene (Figure 4B). These results suggested that the aberrant DNA methylation and imprinting that were established and maintained in early passages (Figure 4C) were not sustainably reversed by the treatment.

## Discussion

Most hES and hiPS cell lines possess stable imprinted gene expression, at least in undifferentiated cells [30,31] and findings in this study]. This implies that imprints withstand the process of reprogramming and the rigors of growing in culture. In our study, we found that only three of the 22 hiPS cell lines we derived from a variety of somatic cell types showed LOI, and at only a few sites. The majority of cases had normal imprinting status. While LOI was rare in our hiPS cell lines, we found that it was maintained during prolonged passage, and resistant to VPA treatment. These abnormalities would preclude the use of these cell lines for therapeutic applications but might provide a mechanistic insight relevant to imprinting and reprogramming.

We previously reported that abnormal DNA methylation detected in early passage iPSCs diminished after continued passaging, such that these cells ultimately more resembled ESCs. However, abnormal DNA methylation at imprinted loci in ESCs occurs in response to continuous passaging [29]. Rugg-Gunn et al. suggested three possible explanations for LOI in hESCs [30]. First, the developmental onset of transcription might influence imprinted gene expression. Second, a particular imprinted gene’s expression might differ depending on whether it is regulated by maternally or paternally inherited methylation. Third, the pattern of imprinted gene expression might depend on whether the gene provides a growth advantage to hESCs. These possibilities might also apply to hiPSCs.

There are two caveats that apply to this work. First, we examined expression in undifferentiated cells. Consequently, we may have missed changes in imprinted gene expression where genes are expressed only in differentiated cells or where imprinting is tissue specific. Second, we examined total levels of expression and total methylation patterns of populations of cells. Therefore we cannot exclude the possibility that a small population within our samples could behave in a different manner from the general population. Nonetheless, our data are encouraging in suggesting that imprinting errors in iPSCs are derived from a variety of human somatic cell types.

One of the key advantages of iPSCs is that they can be derived from patients, supporting the further investigation of certain diseases, as well as the replacement of degenerated and damaged tissues. Careful analysis of imprinted genes should therefore be performed on all iPS cell lines since several published iPS cell lines that passed the necessary reprogramming criteria also showed aberrations in imprinted gene expression and DNA methylation of DMRs. This is particularly critical if these hiPSs are to be used for regenerative medicine since aberrations in imprinted genes could cause problems with cell differentiation and perhaps even cause tumors [38]. The analysis of imprinted genes is also essential for modeling of genetic diseases because abnormal imprinting can seriously confuse the disease phenotyping.

Recent advances in high-throughput technologies for gene expression analysis and DNA methylation analysis indicate the possibility that all newly generated stem cell lines can be characterized at the epigenetic level rapidly and precisely. However, our work and that of others suggest that certain imprinted loci may be more susceptible to LOI. This means that it might be possible to design targeted assays for specific loci as the first step in the characterization of newly generated cell lines, and also those that have been extensively passaged.

## Conclusions

In conclusion, while imprinting errors may be rare in iPSCs, they are resistant to reversal strategies. The aberrant expression of imprinted genes in these lines is likely to hamper their use both for the understanding of certain pathologies and regenerative medicine.

## Methods

### Ethics statement

All experiments handling human cells and tissues were performed in line with the tenets of the Declaration of Helsinki. This study was approved by the Institutional Review Board of the National Institute for Child Health and Development and the Ethics Committee of Tohoku University School of Medicine.

### DNA/RNA preparation of iPSCs

We generated 22 hiPSCs from extraembryonic amniotic membrane (AM-iPS), embryonic lung tissue (MRC-iPS), uterine endometrium (UtE-iPS), adult menstrual blood (Edom-iPS), and extraembryonic placental tissue (PAE-iPS) and characterized the pluripotent nature using culture methods described previously [39-41]. Prior to RNA and DNA preparation, feeder layers were removed from the undifferentiated cells by panning for 20 minutes.

### Loss of heterozygosity (LOH) and loss of imprinting (LOI) analyses

PCR was performed on parental tissue and the genomic DNA of hiPSCs using the primer sequences summarized in Additional file 3. A PCR reaction mix containing 0.5 μM concentrations of each primer set, 200 μM dNTPs, 1× PCR buffer, and 1.25U of EX *Taq* Hot Start DNA Polymerase (Takara Bio, Tokyo, Japan) in a total volume of 20 μl was used. The following PCR program was used: 1 minute of denaturation at 94°C followed by 35 cycles of 30 seconds at 94°C, 30 seconds at 50-70°C, 30 seconds at 72°C and a final extension for 5 minutes at 72°C. PCR products were digested by unique polymorphic enzymes to identify samples that were heterozygous for a single nucleotide polymorphism (SNP). For samples found to be heterozygous for a SNP, RNA was prepared from matched hiPSCs, followed by reverse transcription-PCR (RT-PCR) and restriction digestion (Additional file 3) [42-49]. The digested PCR products were electrophoresed on 3% agarose gel.

### Gene expression analysis

RNA expression levels of 8 imprinted genes were also analyzed by microarray and the real-time PCR. Microarray analysis was performed using an Agilent Whole Human Genome Microarray chip (G4112F, Agilent, Santa Clara, CA). Raw data were normalized and analyzed using GeneSpringGX11 software (Silicon Genetics, Redwood City, CA). The microarray data have been deposited in Gene Expression Omnibus (http://www.ncbi.nlm.nih.gov/geo/). Real-time PCR reaction was done with SYBR Premix Ex Taq II (Takara Bio). In the case of PEG3 expression analysis, TaqMan Gene Expression Assay (Assay ID: Hs00300418-s1, Applied Biosystems, Foster City, CA) was carried out according to the manufacturer’s protocol using a StepOne Real-time PCR System (Applied Biosystems). The relative expression levels of the detected genes from these cells were estimated visually by comparing relative band intensities with the expression level of the housekeeping gene *GAPDH*.

### Polymorphic bisulfite PCR methylation assay

We performed standard methylation assays using the SNPs and bisulfite sequencing [50]. The primary DMRs of eight imprinted genes (*H19, GTL2, ZDBF2, PEG1*, *KCNQ1OT1*, *ZAC*, *PEG3* and *SNRPN*) were analyzed as described previously [50,51]. Each DNA sample was treated with sodium bisulfite using the EZ methylation kit (Zymo Research, Orange, CA) and amplified by PCR. PCR products were purified, cloned into pGEM-T (Promega, Medison, WI) and an average of 20 clones per individual were sequenced using reverse primer M13 and an automated ABI Prism 3130xl Genetic Analyzer (Applied Biosystems). To avoid any allelic bias, we used specific polymorphic sites. Sodium bisulfite modification treatments were carried out in duplicate for each DNA sample and at least three independent amplification experiments were performed for each individual examined.

#### Chromatin immunoprecipitation (ChIP) assay

ChIP analysis was performed using the Magna ChIP G Chromatin Immunoprecipitation Kit (Millipore, Temecula, CA) according to the manufacturer’s protocol. We used the following antibodies: dimethylated H3-Lys4, acetylated H3-Lys9, dimethylated H3-Lys9 and trimethylated H3-Lys27 (Millipore). The histone modifications were analyzed by real-time PCR. Real-time PCR reaction was done with SYBR Premix Ex Taq II (Takara Bio). The amount of precipitated DNA was determined as percentage relative to input DNA. Primers used are listed in Additional file 3.

#### Treatment of cells with sodium valproate

hiPSCs were plated at a density of 5 × 10^5^ cells/60mm^2^ dish. Twenty-four hours later, they were treated with 1 mM sodium valproate (Wako, Tokyo, Japan) for the times stated. Total RNA was prepared and analyzed by the RT-PCR method. The methylation status of the DMRs was examined using the bisulfite PCR sequencing methylation assay described previously [51].

## Abbreviations

DMR: Differentially methylated region; hESCs: Human embryonic stem cells; hiPSCs: Human induced pluripotent cells; LOH: Loss of heterozygosity; LOI: Loss of imprinting; MOI: Maintenance of imprinting; N.D: Not determined; N.T: Not tested; PCR: Polymerase chain reaction; RFLP: Restriction fragment length polymorphism; RT-PCR: Reverse transcription-PCR; SNP: Single nucleotide polymorphism; SD: Standard deviation.

## Competing interest

The authors declare that they have no competing interests.

## Authors’ contributions

AU and TA conceived and designed the study and wrote the manuscript. AU generated hiPSCs and hESCs. TA analyzed genomic imprinting. HH carried out the molecular study and data analysis and wrote the manuscript. H Okae, MS, NM and AS performed the molecular study and contributed to data analysis. MT, NK, H Okita, YM and HA prepared cell materials and contributed to data analysis. KN performed the transcriptome data analysis. All authors reviewed the results from the data analysis and approved the final manuscript.

## Supplementary Material

Additional file 1 **Microarray analysis.** Scatter plots of MRC-iPS-25P22 versus hES3 (**A**) and UtE-iPS-6P30 versus hES3 (**B**). Scatter plot comparing the spot intensities in hybridization with probes from hiPSCs (*y* axis) and hESCs (*x* axis). The magenta plots indicate the imprinted genes. Click here for file

Additional file 2 **Gene expression analysis of the imprinted genes.***H19* (**A**), *IGF2* (**B**), *PEG3* (**C**), *PEG1* (**D**), *GTL2* (**E**), *KCNQ1* (**F**), *NDN* (**G**) and *LIT1* (**H**). Gene expression of the original and hiPSCs was compared to that of hESCs. The *GAPDH* ratio was calculated. The bars indicate the means ± SD from two replicates. Click here for file

Additional file 3 PCR primers and conditions. Click here for file
