# Supplementary figures and images for: Stability of genomic imprinting in human induced pluripotent stem cells
Source: BMC Genet. 2013 Apr 30;14:32. doi: 10.1186/1471-2156-14-32 (PMC3751563; doi:10.1186/1471-2156-14-32)

## Slide 1
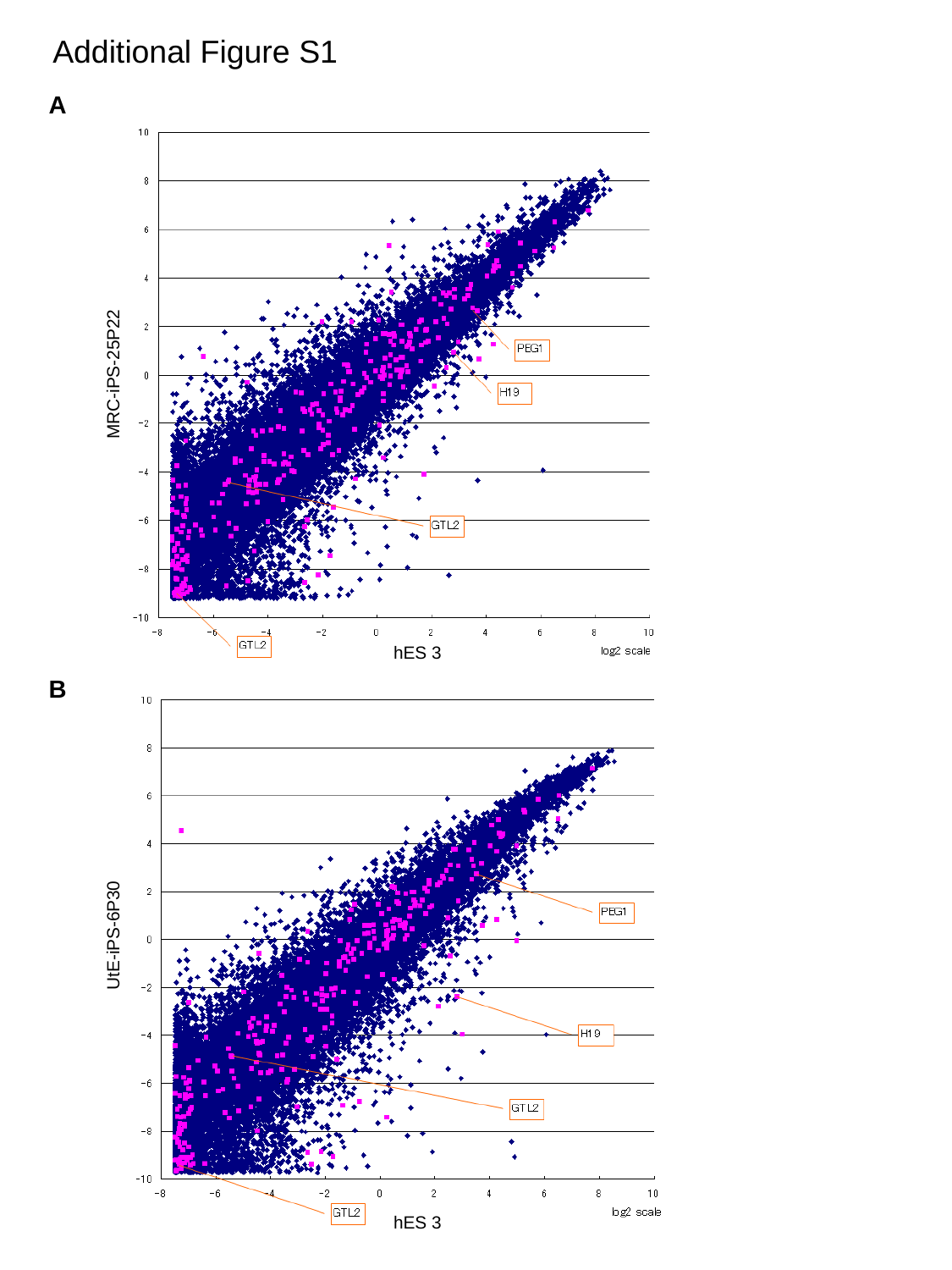

Additional Figure S1
A
MRC-iPS-25P22
hES 3
B
UtE-iPS-6P30
hES 3

Supplement: Additional file 1 — Microarray analysis. Scatter plots of MRC-iPS-25P22 versus hES3 (A) and UtE-iPS-6P30 versus hES3 (B). Scatter plot comparing the spot intensities in hybridization with probes from hiPSCs (y axis) and hESCs (x axis). The magenta plots indicate the imprinted genes. [file 1471-2156-14-32-S1.ppt]
