# Supplementary material for: Stability of genomic imprinting in human induced pluripotent stem cells
Source: BMC Genet. 2013 Apr 30;14:32. doi: 10.1186/1471-2156-14-32 (PMC3751563; doi:10.1186/1471-2156-14-32)
Supplement: Supplementary file 2 [file 1471-2156-14-32-S3.doc]

Additional file 3. PCR primers and conditions.

Gene	Target		Primer sequence (5'-3')	Amplicon (bp)	annealing	Accession No.	Enzyme
H19	gDNA	H19 RTF1	TGCTGCACTTTACAACCACTG	635	66	AF125183	*Rsa*I
		H19 RTR2	GTCGGAGCTTCCAGACTAGG				
	cDNA	H19 RTF1	TGCTGCACTTTACAACCACTG	554	66		
		H19 RTR2	GTCGGAGCTTCCAGACTAGG				
	qPCR	hH19 exqF1	GCAAGAAGCGGGTCTGTTT	105	60	NR_002196	
		hH19 exqR1	GCTGGGTAGCACCATTTCTT				
GTL2	gDNA	GTL2 F6	GTGTGTACCTTGGTTGGTGACTC	368	66	AL117190	*Taa*I
		GTL2 R2	GAGGCATATATTTGAGTTACACATACCCCTTAGTCC				
	cDNA	GTL2 F6	GTGTGTACCTTGGTTGGTGACTC	368	66		
		GTL2 R2	GAGGCATATATTTGAGTTACACATACCCCTTAGTCC				
	qPCR	hGTL2 exqF1	CTCGTCTCCTTCCTGGTTTG	122	60		
		hGTL2 exqR1	AGGGCACTTCCATCCTAGTCT				
	Bisulfite	GTL2 F	GGGTTGGGTTTTGTTAGTTGTT	459	57		*Taq*I
		GTL2 R2 SNP	ACAATTTAACAACAACTTTCCTCCA				
	ChIP	GTL2 C1F2	CGGGCATTTTATTTTCCTGAACAATA	84	60		
		GTL2 C1R2	GGTTTCGGAACATCCATCGGAGG				
IGF2	gDNA	IGF-II IP2	TTGAGTCAAATTGGCCTGGA	279	60	AF517226	*Apa*I
		IGF-II IP3	TGCCAATTACATTTCATTTGC				
	cDNA	IGF-II IP2	TTGAGTCAAATTGGCCTGGA	279	60		
		IGF-II IP3	TGCCAATTACATTTCATTTGC				
	qPCR	hIGF2 exqF1	ACACCCTCCAGTTCGTCTGT	101	60	NM_001007139	
		hIGF2 exqR1	GAAACAGCACTCCTCAACGA				
PEG3	gDNA	P3-MnlI-F	CCCTCCCCTCGCATAATAACTA	339	60	AC006115	*Mnl*I
		P3-MnlI-R	TCTTCTGTCTGTCTCCTCTCCC				
	cDNA	P3-MnlI-F	CCCTCCCCTCGCATAATAACTA	339	60		
		P3-MnlI-R	TCTTCTGTCTGTCTCCTCTCCC				
PEG1	gDNA	PEG1 PegF	TACTAAACCAGCATACCCTTAC	308	66	AB045582	*Afl*III
		PEG1 PegR	GCAGTCATCATAAAGGAATCAG				
	cDNA	PEG1 PegF	TACTAAACCAGCATACCCTTAC	308	66		
		PEG1 PegR	GCAGTCATCATAAAGGAATCAG				
	qPCR	hPEG1 exqF1	GACGGGAACTTAGTCATTGACAG	125	60	NM_002402	
		hPEG1 exqR1	TCCAATGGCCCATAGATAAAA				
	Bisulfite	PEG1 BSF1	AATTTTAATTATTTGATGAGTTATGAG	275	57	AB045582	*Nru*I
		PEG1 BSR1	ATATTTTTCAAATTTCAATAACAAAC				
KCNQ1	gDNA	hKCNQ1-13 SF	GCTGGGGCACAGGGAGGAGAAGTG	175	70	NC_000011	*Sma*I
		hKCNQ1-13 SR	GCACCCTCCCCGCTGCCGTTTG				
	cDNA	hKCNQ1-13 exSF2	CATCACCCACATCTCACAGC	221	65	NM_000218	
		hKCNQ1-13 exSR2	ACAGTGAGGGCTTCCCAAT				
	qPCR	hKCNQ1 exqF1	CATCACCCACATCTCACAGC	73	60		
		hKCNQ1 exqR1	AAAGTACTGCATGCGTCGAA				
KCNQ1OT1/LIT1	gDNA	LIT1 SMS2-B	AGGAAAGTGTTGAGTGGTAA	803	60	NR_002728	*Rsa*I
		LIT1 SMS2-C	CAAGCAGTACTGTTTCAGAA				
	cDNA	Lit102	TCTGGTTCATGTCACTCTGTGGAGCAG	477	50		
		Lit202	CTCCCAAAAGCAGAGTTTTGGCAATAT				
	qPCR	hLIT1 exqF1	CCCATGGACATCCCTTACAC	75	62		
		hLIT1 exqR1	GCCAATGGCTCATGCTTT				
NDN	gDNA	NDN NEC20F	GCCCGAATACGAGTTCTTTT	540	66	NM_002487	*Mbo*I
		NDN NEC6R	CACACATCATCAGTCCCATA				
	cDNA	NDN NEC20F	GCCCGAATACGAGTTCTTTT	540	66		
		NDN NEC6R	CACACATCATCAGTCCCATA				
	qPCR	hNDN exqF1	CTGATGATGTGTGTTGGGGTA	62	60		
		hNDN exqR1	GGCTTTGCTGGTGACTTCTT				
GAPDH	qPCR	GAPDH exF	GTCGTGGAGTCCACTGGCGTC	244	60	NM_002046	
		GAPDH exR	TCATGAGTCCTTCCACGATAC				
